# Editorial: Expanding CAR-T cell therapy — breakthroughs from cancer to autoimmune diseases

**DOI:** 10.3389/fimmu.2025.1649045

**Published:** 2025-08-01

**Authors:** Selene Nunez-Cruz, Yibo Yin, Jianlei Hao, Hongru Zhang

**Affiliations:** ^1^ Department of Pathology and Lab Medicine, Perelman School of Medicine, University of Pennsylvania, Philadelphia, PA, United States; ^2^ Center for Cellular Immunotherapies, Perelman School of Medicine, University of Pennsylvania, Philadelphia, PA, United States; ^3^ Department of Neurosurgery, The First Affiliated Hospital of Harbin Medical University, Harbin, China; ^4^ The Biomedical Translational Research Institute, Faculty of Medical Science, Jinan University, Guangzhou, Guangdong, China; ^5^ College of Life Sciences, Nankai University, Tianjin, China

**Keywords:** cancer, autoimmune disease, CAR T, CRISPR, toxicity

Since the FDA’s landmark approval of the first Chimeric antigen receptor T-cell (CAR-T) cell therapy in 2017, the landscape of tumor immunotherapy has undergone a profound transformation. CAR-T therapy has revolutionized cancer treatment, particularly for hematologic malignancies, with six CAR-T cell therapies now FDA-approved. These advances have not only improved survival for patients with otherwise treatment-resistant cancers but have also sparked investigations into broader applications, including autoimmune diseases and other non-malignant conditions.

## Summarize of this Research Topic

This Research Topic in Frontiers in Immunology (Expanding CAR-T Cell Therapy: Breakthroughs from Cancer to Autoimmune Diseases) brings together seven impactful contributions—three original research articles and four reviews—that collectively underscore the rapid evolution and translational potential of Chimeric Antigen Receptor (CAR) T cell therapy in both cancer and autoimmune disease.

The original studies present significant advances in CAR-T engineering. Cook et al. introduce ARMed CAR-T cells, which incorporate checkpoint antibody receptors to overcome the highly immunosuppressive microenvironment of glioblastoma, demonstrating promising efficacy (In press). Zeng et al. optimize gene transfer with a low-CpG Passer transposon, yielding superior CAR-T persistence and functionality. Meanwhile, Wei et al. engineer T cell Antigen Coupler -T(TAC-T) cells targeting NECTIN-4, revealing enhanced antitumor effects against solid tumors via CD28 co-stimulation.

The accompanying reviews provide comprehensive perspectives on expanding the CAR-T landscape. Cheever et al. explore novel CAR technologies aimed at treating autoimmune diseases, emphasizing antigen-specific immune regulation. Tang et al. propose a strategy combining FcγRI-expressing T lymphocytes with monoclonal antibodies, offering new options for refractory cancers. Niu et al. analyze the current progress and unique challenges of CAR-T therapy in breast cancer, while Huang et al. highlight non-conventional sources of CAR products, paving new paths for innovation in immunotherapy.

## CAR-T in hematologic malignancies and beyond

Anti-CD19 CAR-T therapies have emerged as among the most successful interventions for B-cell malignancies. Their clinical efficacy has redefined outcomes for diseases such as acute lymphoblastic leukemia and large B-cell lymphoma. Recently, this success has extended beyond oncology (1). Notably, anti-CD19 CAR-T cells have demonstrated clinical benefit in refractory systemic lupus erythematosus (SLE), suggesting the immune-modulatory power of CAR-T cell therapy can be harnessed for autoimmune disorders (2).

The expanding body of clinical data continues to support the notion that CAR-T cells, originally developed for cancer, may offer new therapeutic solutions for otherwise intractable autoimmune diseases. This marks a pivotal shift from the therapy’s oncologic origins to broader immunologic applications (3).

## Technological advances driving the CAR-T evolution

Despite early successes, traditional CAR-T therapies face several limitations, particularly in treating solid tumors. Tumor heterogeneity, an immunosuppressive microenvironment, and T-cell exhaustion hinder therapeutic efficacy (4). To overcome these challenges, new strategies are emerging to enhance CAR-T cell precision, persistence, and safety.

- Dual-target CARs aim to reduce antigen escape by enabling CAR-T cells to recognize two distinct tumor antigens. This ensures therapeutic efficacy even if one antigen is downregulated (5).
- SynNotch CARs, engineered to respond to environmental cues in the tumor microenvironment, allow for more context-specific activation, reducing off-tumor effects (6).
- iCARs (inducible CARs) introduce a regulatory switch, enabling external control of CAR-T activation and minimizing unintended immune responses (7).

Further innovations include chemokine receptor-engineered CAR-T cells for improved trafficking to solid tumors and antibody-secreting CAR-T cells that can recruit endogenous immune responses. Together, these refinements aim to address tumor evasion and immune suppression, which have historically limited CAR-T success in non-hematologic cancers.

## A new frontier: CAR-T for autoimmune diseases

The application of CAR-T cell therapy to autoimmunity represents one of the most exciting and fast-growing areas of immunotherapy. Autoimmune diseases are marked by immune dysregulation and self-reactivity. By selectively eliminating autoreactive immune cells, CAR-T cells offer a targeted method of re-establishing immune tolerance.

Preclinical and early clinical data support this approach. For example:

- Anti-DSG3 CAR-T cells have shown potential in pemphigus vulgaris, a rare autoimmune skin disorder (8).
- Anti-MuSK CAR-T cells are under investigation for autoimmune myasthenia gravis (9).

These therapies represent a paradigm shift: from global immunosuppression to targeted immune correction. Unlike conventional treatments, which often carry broad and chronic immunosuppressive risks, CAR-T cells offer the potential for long-lasting remission with a single intervention.

## Key challenges in CAR-T cell therapy

While transformative, CAR-T therapy still faces important obstacles:

1. Antigen Escape: Tumors may downregulate or lose the targeted antigen, rendering CAR-T cells ineffective. This is particularly problematic in solid tumors, where antigen expression is less uniform. Dual-target and bispecific CAR-T cells offer a promising countermeasure (10).
2. Cytokine Release Syndrome (CRS): A potentially severe inflammatory response, CRS remains a major toxicity concern. Strategies such as safety switches (iCARs) and refined dosing regimens are being developed to mitigate this risk (11).
3. On-target Off-tumor Toxicity: Target antigens may be present on normal tissues, leading to collateral damage. To enhance specificity, researchers are designing CARs that respond to combinations of antigens or to microenvironment-specific cues, thereby sparing healthy cells (12).
4. Persistence and Exhaustion: Durable responses depend on CAR-T cells that persist without becoming functionally exhausted. Efforts to enhance memory phenotypes and resist inhibitory signals in the tumor microenvironment are central to next-generation CAR-T designs (13).

## Future directions and clinical integration

The future of CAR-T cell therapy lies in refining its engineering and expanding its indications. Technological innovations such as non-viral gene delivery, CRISPR-based genome editing, and site-specific insertion techniques are improving the safety, efficiency, and scalability of CAR-T manufacturing.

Equally important is the discovery of novel disease-specific targets—both for cancers and autoimmune diseases. Identifying antigens uniquely expressed in diseased tissues will be key to minimizing toxicity and enhancing therapeutic index (14).

CAR-T cell therapy is also poised to benefit from combination strategies (15). For instance, pairing CAR-T cells with immune checkpoint inhibitors or tumor-penetrating agents may enhance efficacy in solid tumors. In autoimmunity, integrating CAR-T with transient immunomodulation could help create a more permissive environment for immune reset.

## Conclusion

This special Research Topic in *Frontiers in Immunology* provides a timely overview of the latest advances in CAR-T cell therapy and its expanding horizon. From hematologic malignancies to autoimmune diseases, CAR-T technology is evolving rapidly—powered by engineering innovations, new therapeutic targets, and a deepening understanding of immune biology.

While challenges such as antigen escape, toxicity, and limited efficacy in solid tumors remain, the solutions under development are equally promising. The success of CAR-T cell therapy in refractory SLE and its potential in other autoimmune disorders signals a new era of targeted, durable immunotherapy.

As the field advances, CAR-T cell therapy stands at the forefront of personalized medicine, offering renewed hope for patients facing diseases that once had few or no effective treatments. With continued interdisciplinary collaboration and innovation, CAR-T cells may soon become a cornerstone of therapy well beyond oncology.
